# Deciphering the effects of genotype and climatic factors on the performance, active ingredients and rhizosphere soil properties of *Salvia miltiorrhiza*

**DOI:** 10.3389/fpls.2023.1110860

**Published:** 2023-04-20

**Authors:** Chao He, Tingting Han, Chang Liu, Peng Sun, Dengqun Liao, Xianen Li

**Affiliations:** Institute of Medicinal Plant Development, Chinese Academy of Medical Sciences & Peking Union Medical College, Beijing, China

**Keywords:** *Salvia miltiorrhiza*, plant performance, active compounds, climate, soil physicochemical properties, soil microbial composition, genotype

## Abstract

**Introduction:**

*Salvia miltiorrhiza* Bunge is an important medicinal herb, which is widely cultivated in most parts of China. It has attracted considerable attention because of its pharmacological properties and potential health benefits.

**Methods:**

We used a field experiment to determine the effects of different genotypes and climatic factors on the performance (plant biomass, morphological parameters), active ingredients, rhizosphere soil physicochemical properties and microbial composition of *S. miltiorrhiza* at five cultivation locations.

**Results:**

The results showed that these parameters were significantly different in the six different genotypes of *S. miltiorrhiza* from five producing areas. Genotype and soil physicochemical properties were the main factors affecting the growth traits of *S. miltiorrhiza*, while genotype, climate and soil physicochemical properties were the main factors affecting the content of active components of *S. miltiorrhiza*. Microbial phospholipid fatty acid analysis showed that the biomass of Gram-positive and Gram-negative bacteria was affected by the genotypes of *S. miltiorrhiza* plants, while the biomass of arbuscular mycorrhizal fungi, fungi, Gram-positive and Gram-negative bacteria was affected by climate factors.

**Discussion:**

Based on the main results, DS993 was the most suitable genotype for *S. miltiorrhiza* in the five producing areas from the perspective of comprehensive growth traits and medicinal components, while DS993 and DS2000 were suitable for planting in Shandong province from the perspective of origin. DS996 is not suitable for all of the above production areas. These results are helpful to understand the ecological adaptability of different genotypes of *S. miltiorrhiza* resources, and to select appropriate S. miltiorrhiza genotypes for specific planting areas, so as to maximize yield and quality.

## Introduction

Medicinal plants are important sources of active ingredients that affect human health, and their raw materials are widely used in the pharmaceutical industry (Weckesser et al., 2007). According to a World Health Organization report, plants and their compounds can replace chemical drugs and have fewer side effects (Zargoosh et al., 2019). The performance and accumulation of the active ingredients in medicinal plants are governed by both genetic and environmental factors. The ancient Chinese ‘geoherbalism’ theory indicated that there would be variation in the quality of medicinal materials from different regions (Li et al., 2012). The performance and active components will, therefore, show interspecific, intraspecific and ontogenic variations, as well as variations associated with climate and soil conditions. Medeiros Melo et al. (2019) and Ma et al. (2020) considered that the contents and compositions of the active ingredients in *Pacupevas* and *Prickly ash* plants were widely affected by plant genotype, management measures, climatic and soil conditions. Aboukhalid et al. (2017) found that *Origanum compactum* of the same species growing in different environments may show differences in the composition of their essential oils. The growth, development, reproduction, behavior and distribution of medicinal plants are not only guided by genetic factors, but also directly or indirectly influenced by environmental factors, including climate, geography and soil type (Yuan et al., 2020; Miransari et al., 2022). Recently, studies concerning the effects of a wide range of climatic conditions and edaphic factors on the content of active ingredients in medicinal herbs from different geographical locations have been carried out (Liu et al., 2015; Pant et al., 2021). Climatic factors such as temperature, precipitation, humidity and sunshine duration, regulate the active ingredient contents in medicinal plants and markedly influence the morphological structure, physiological characteristics and chemical composition (Zobayed et al., 2005; Patni et al., 2022). Moreover, the distribution characteristics of the content and proportion of plant effective components were also affected by the topography and soil properties of the cultivation site (Marsol-Vall et al., 2018). Therefore, soil factors, climatic factors, topography of growing location and orientation of both sunny and negative slopes are the main environmental conditions that affect the selection of suitable planting areas for medicinal plants.

In natural environments without human intervention, medicinal plants grow in soil, so different aspects of their growth and development and accumulation of active ingredients are heavily influenced by soil factors such as nutrient content and microorganisms (Cheng et al., 2014; Zargoosh et al., 2019; Ma et al., 2020). These factors play a very important role in the production of metabolites, and the root system is the main channel transferring soil nutrients to the plant’s different parts (Zhang et al., 2018; Ramos and de Toda, 2019). In addition, soil microorganisms are the most active part of the soil, and are the driving force of soil material transformation and nutrient cycling. Soil microorganisms participate in various soil processes, such as decomposition of soil organic matter, and the formation of humus, as well as the transformation and circulation of soil nutrients. The quantity and species of rhizospheric soil microbes have been consistently shown to be important factors affecting the growth, development and health of plants (Li et al., 2018; Dang et al., 2021). For example, some microbes in the soil are often the leading cause of plant diseases (Bian et al., 2020), while beneficial soil microbes are crucial for plant growth and accumulation of active components (Zhai et al., 2018; Piszczek et al., 2019; Dang et al., 2021).

*Salvia miltiorrhiza* Bunge (Lamiaceae) is an important medicinal plant in traditional Chinese medicine. Its dried roots and rhizomes have been used for the treatment of heart diseases including myocardial infarction, cerebrovascular diseases, and Alzheimer’s disease (Zhou et al., 2021). The major active compounds in *S. miltiorrhiza*, water-soluble components (rosmarinic acid, and salvianolic acid B) and lipid-soluble components (tanshinone I, tanshinone ιI A, and cryptotanshinone), which are mainly accumulated in the roots and rhizomes (Yang et al., 2016). Compound *S.miltiorrhiza* Dripping pills (DSP), one of the traditional Chinese medicine preparations, has passed Phase II clinical trials of the US Food and Drug Administration for its remarkable efficacy in treating cardiovascular diseases.

Although *S.miltiorrhiza* is widely distributed in China and has a long history of cultivation, the interaction mechanism of different genotypes and complex environmental factors on plant growth and accumulation of effective components remains unclear. Zhong et al. (2009) found that different geographical origins of *S. miltiorrhiza* samples might lead to differences in the primary and secondary metabolites. Zhao et al. (2016) reported that the same genotypes grown in different places and different genotypes grown in the same place showed significant variation in their metabolome, and the content of bioactive components was affected by the growing environment and genotype. Zhang et al. (2018) investigated the effects of environmental and soil parameters on secondary metabolite contents in *S. miltiorrhiza* from different natural habitats of China. Liu et al. (2022) reported that cultivation location clearly regulated the composition of *S. miltiorrhiza* samples. Since many potential factors affect the growth and distribution of medicinal plants, it is necessary to evaluate multiple mechanisms simultaneously in order to understand their relative importance.

In the current study, six different genotypes of *S.miltiorrhiza* were planted in five different ecological experimental stations to determine the combined effects of genotype, climatic conditions and soil properties on the general performance and effective composition of *S.miltiorrhiza* We speculate that the growth and development process of *S.miltiorrhiza* is not limited to a single factor, but is influenced by a variety of complex factors from inside to outside. Therefore, when analyzing the growth and accumulation of active components of *S. miltiorrhiza*, we should consider the plant and its living environment as a whole, and analyze it from different perspectives such as genotype, climate, soil physicochemical properties and soil microorganisms. Only in this way can we more comprehensively reveal the essence and mystery of the growth and metabolic activities of *S.miltiorrhiza*. It can provide scientific guidance for subsequent screening of suitable *S.miltiorrhiza* genotypes in different producing areas.

## Materials and methods

### Field experiment design and sample collection

We planted six genotypes of *S.miltiorrhiza* (DS2000, DS992, DS993, DS994, DS995, DS996) in Beijing, Anhui, Shandong, Shaanxi and Sichuan respectively (the experimental arrangement and management methods were consistent in all planting areas) (**Tables 1** and **2**).The total site area of each planting area was 240 m^2^, among which the experimental plot area was 170 m^2^. All the experiments were arranged according to the randomized block method. A total of 6 genotypes of *S.miltiorrhiza* planting treatments were set up in the experiment, and 3 repeated test plots of 9 m^2^ (3 m×3 m) were set up in each treatment, and a total of 18 test plots were set up in 6 genotypes. Four ridges 35 cm high and 25 cm wide were set up in each plot. One row of *S.miltiorrhiza* was planted on each ridge, and there were four rows of *S.miltiorrhiza* in each experimental field.

**Table 1:** Genotype sources and characters of different genotypes of *Salvia miltiorrhiza*.

Genotype number	Genotype source	Genotype character
DS2000	Zhongjiang County, Sichuan Province	Leaves are smaller. Flowers are purple. Roots are thick and red.
DS992	Fangcheng County, Henan Province	Leaf edge teeth deep ccenter curl, leaf surface fold obvious, dark color. The leaves are purple at spring bud. The flowers are purple and dark in color. Root is purplish red with fine texture.
DS993	Juxian County, Shandong Province	Leaves are larger. Flowers are purple, less flowering. Roots are rough and red.
DS994	Juxian County, Shandong Province	Leaves are smaller. Flowers are purple. Red roots, medium size.
DS995	Tongchuan City, Shaanxi Province	Leaves are larger. Flowers are purple. Roots are red and thicker.
DS996	Quanjiao County, Anhui Province	Leaf blade small, rounded, light color. Flowers are purple and white, and the flowering period is longer. Roots are red and small.

**Table 2:** Information of cultivation sites and soil physicochemical property in different producing areas.

Planting site	Local information	pH	Soil organic matter(g/kg)	Available N(g/kg)	Available P(g/kg)	Available K(g/kg)
Haidian District, Beijing	N 42°15′, E 116°12′	7.72 ± 0.12b	10.21 ± 0.67a	0.81 ± 0.06b	0.66 ± 0.02b	17.62 ± 0.10c
Bozhou, Anhui Province	N 34°02′, E 117°23′	7.86 ± 0.38b	8.75 ± 0.89b	0.68 ± 0.09c	0.84 ± 0.06a	19.17 ± 0.09b
Juxian, Shandong Province	N 36°30′, E 118°41′	7.61 ± 0.26b	10.23 ± 0.35a	0.83 ± 0.12b	0.77 ± 0.03a	23.05 ± 0.12a
Shangluo, Shaanxi Province	N 34°18′, E 110°05′	8.15 ± 0.19a	9.57 ± 0.94ab	1.05 ± 0.07a	0.81 ± 0.08a	15.36 ± 0.16d
Zhongjiang, Sichuan Province	N 31°51′, E 105°38′	6.89 ± 0.22c	7.41 ± 0.42c	0.74 ± 0.12bc	0.62 ± 0.04b	16.60 ± 0.07cd

Different letters in the same column indicate significant differences between different samples sites (P < 0.05).

On March 6, 2019, seeds of six genotypes were planted in five planting areas. On November 5, 2019, the six genotypes of *S.miltiorrhiza* and the rhizosphere soil in each planting area were sampled using a unified method. The sampling method of Beijing is taken as the standard to elaborate in detail., 15 healthy *S.miltiorrhiza* plants were collected from three different repeated plots of each *S.miltiorrhiza* genotype treatment (5 plants were collected from each experimental plot), so a total of 90 samples were collected from the six genotypes of *S.miltiorrhiza*.Then the samples of *S.miltiorrhiza* plants were packed and numbered with different experimental plots of different genotypes as taxonomic units (for example, Beijing1 (DS2000), Beijing2 (DS2000), Beijing3 (DS2000), Beijing1 (DS992), Beijing2 (DS992), Beijing3 (DS992)…). The method of collecting rhizosphere soil is to gently shake the soil attached to the root and put it into a plastic bag as rhizosphere soil (about 100g). Finally, the rhizosphere soil samples were numbered (consistent with the plant sample number), placed in an insulated container, and then transported to the laboratory for analysis.The collection method of plant and rhizosphere soil samples from other production areas is the same as that of Beijing.

### Climate parameters

An automatic weather station was set up at each experimental site. From the start of the experiment until the harvest, the station recorded hourly data on air humidity, temperature, optical radiation and rainfall. In addition, the temperature and water content of 10 cm soil were also measured. After the experiment, the data from each experimental site were collected and analyzed (**Table 3**).

**Table 3:** Climatic factors at different sampling sites.

Sampling sites	Average temperature (°C)	Mean temperature difference (°C)	Soil mean temperature (°C)	Mean rainfall (mm)
Beijing	23.4 ± 0.57b	20.4 ± 0.72ab	22.8 ± 0.68ab	165.7 ± 12.97a
Anhui	25.1 ± 0.35a	21.6 ± 0.86a	23.6 ± 0.81a	118.9 ± 10.25c
Shandong	24.5 ± 0.43ab	19.9 ± 0.55ab	23.2 ± 0.77a	148.1 ± 8.99b
Shaanxi	23.6 ± 0.30b	22.1 ± 0.76a	23.3 ± 0.42a	61.75 ± 15.06d
Sichuan	22.9 ± 0.48b	18.1 ± 0.49ab	20.8 ± 0.46b	98.78 ± 13.95c

Different letters in the same column indicate significant differences between different samples sites (P < 0.05).

### Growth parameters of *S. miltiorrhiza*

At harvest, the plant height was recorded, and then the shoots and roots from each plant were harvested separately. The root system of each plant was gently washed to remove any adhering sandy soil. Cleaned root samples were placed in clear plexiglass trays containing deionized water and an image recorded by a scanner (EPSON Perfection V800 Photo, Japan). The mean root diameter and number of root branches were measured using the Win-RHIZO image analysis system (Regent Instruments, Quebec, QC, Canada). The remaining roots and shoots were dried at 80°C for 48 h to calculate the plant biomass.

### Active ingredients of *S. miltiorrhiza*

The contents of water-soluble components (rosmarinic acid, and salvianolic acid B) and lipid-soluble components (tanshinone I, tanshinone ιI A, and cryptotanshinone) in *S. miltiorrhiza* roots were determined using a high-performance liquid chromatography (HPLC). Samples and standards were prepared in accordance with the guidelines of the Chinese Pharmacopoeia. Tanshinone ι, tanshinone ιIA, cryptotanshinone, rosmarinic acid and salvianolic acid B were purchased from the Beijing National Institute of Food and Drug Administration. Working parameters for the determination of water-soluble components were as follows: the column was a Phenomenex Gemini C^18^ column (250 × 4.6 mm, 5µm, Guangzhou Philomen Scientific Instruments Co., Ltd.); the mobile phases were water-acetonitrile-formic acid (90:10:0.4) (phase A) and acetonitrile (phase B), with gradient elution: phase A: 0–40 min, 70%–100%, phase B: 0–40 min, 0%-30%; the detection wavelength was 280 nm; the flow rate was 1 mL/min; the column temperature was 25°C; the injection volume was 20 mL. Working parameters for the determination of liposoluble components were as follows: the column was a Welchrom™ C^18^ column (Analytical 4:6 × 250 mm, 5 µm, Welch Corporation, USA); the mobile phases were methanol (A phase) and water (B phase), with gradient elution: A phase: 0–25 min, 67%–67%; 25–45 min, 67%–90%, B phase: 0–25 min, 33%–33%; 25–45 min, 33%–10%. The detection wavelength was 270 nm; the flow rate was 1 mL/min; the column temperature was 25°C; the injection volume was 20 mL (Zhang et al., 2010).

### Physicochemical properties of *S. miltiorrhiza* rhizosphere soil

Rhizosphere soil samples from *S. miltiorrhiza* were screened with a 2 mm sieve and divided into two sub-samples. One subsample was dried at room temperature for soil physicochemical analyses, and the other was frozen at 80°C for microbial composition analysis. Soil sand content was measured by sieve analysis (Bao, 2003). Soil pH was determined with a digital pH meter (PHS-3C) in a 1:2.5 soil: water suspension.

A dried soil sample (0.2 g) was digested in 10 mL of a mixture containing perchloric acid (12.7 mol/L), sulfuric acid (18 mol/L), and water in the ratio 10:1:2 using the Mars 6 microwave reaction system (CEM Corporation, Matthews, NC, United States) until a clear liquid was obtained. The contents of soil organic matter, available nitrogen (N), available phosphorus (P), and available potassium (K) were quantified using oxidization with dichromate in the presence of sulfuric acid (Rowell, 1994), alkaline hydrolysis diffusion, and the chlorostannous-reduced molybdophosphoric blue method (Olsen et al., 1954) with a flame photometer (Jackson, 1973). Soil urease (U) activity was determined using the method of Hoffmann and Teicher (1961) and expressed as μg of NH^4+^-N released from 1 g of soil over a period of 3 h. Soil acid phosphatase and alkaline phosphatase were detected following the method of Tarafdar and Marschner (1994), and the unit of phosphatase activity was mmol P-nitrophenyl phosphate/g soil/h released by the phosphatase.

### Microbial biomass of *S. miltiorrhiza* rhizosphere soil

Soil microbial composition was determined by analyzing the ester-linked phospholipid fatty acid (PLFA) composition of the soil. About 8.0 g of frozen soil was weighed, and lipids were extracted overnight by the modified Bossio and Scow (1998) method, using 23 mL of chloroform: methanol: phosphate buffer (1:2:0.8 v/v/v) solution. The extracts were separated on silica acid columns by sequential elution using organic solvents with increasing polarity, followed by evaporation under N_2_. Phospholipids were sequentially saponified and methylated to form fatty acid methyl esters. Individual fatty acid methyl esters were identified and quantified using a gas chromatograph (Agilent 6890N) equipped with the MIDI software package Sherlock MIS version 4.5 (MIDI Inc., Newark, Delaware, USA), and PLFA was analyzed. The MIDI package automatically controlled all gas chromatography operations, including calibration, subsequent sample sequencing, peak integration, and nomenclature. The calibration standards contained a mixture of linear saturated methyl hydroxy fatty acid esters with a length of 10–20 carbons (MIDI Part No. 1208) (He et al., 2019).

### Statistical analysis

All statistical analyses were performed using SPSS 21.0 (SPSS Inc., Chicago, IL, USA). All boxplots show individual data points, medians, interquartile ranges, and minimum and maximum values. Three-way ANOVA was used to analyze the effects of genotypes, climate and their interactions on growth, active components, soil physicochemical properties and soil microbial biomass. The statistical significance of the results was tested by Duncan’s multiple comparison test (*P*<0.05). Variance decomposition quantified the contribution of different factors to the growth parameters and active components of *S. miltiorrhiza*. The RStudio software package vegan (Borcard et al., 2011) and Kaleida Graph 4.5 software were used for statistical analysis and graphing. The Mantel test and structural equation modeling (SEM) analysis of climate, genotypes, soil physicochemical properties and soil microbe influences were performed using the R-3.2.2 software package (Goslee and Urban, 2007) and AMOS 21.0 (maximum likelihood) for effects on the growth parameters and active ingredient content of *S. miltiorrhiza*.

## Results

### Plant biomass and growth parameters

**Figure 1:**
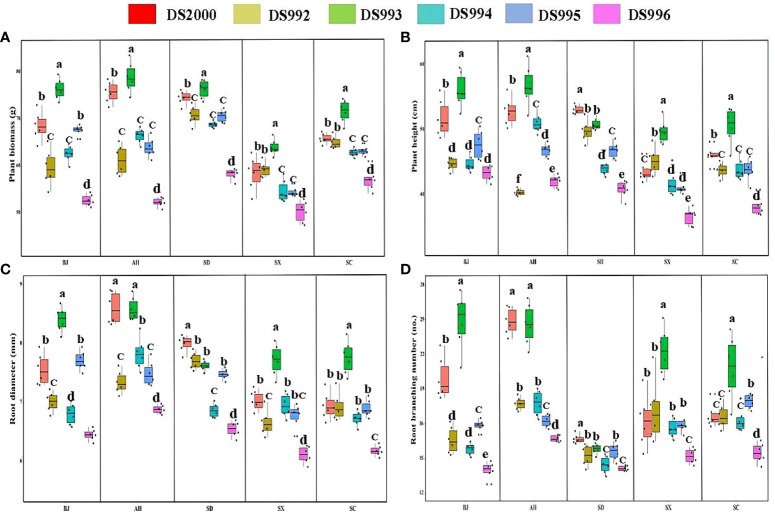
Plant growth parameters of *Salvia miltiorrhiza* genotype in different cultivation locations. Plant biomass **(A)**; Plant height **(B)**; Root diameter **(C)**; Root branching number **(D)**. BJ, Beijing; AH, Anhui; SD, Shandong; SX, Shaanxi; SC, Sichuan. Different letters above the error bars indicate significant difference at *P* < 0.05.

Three-way ANOVA results showed that genotypes significantly affected plant growth parameters, climatic factors significantly affected plant biomass and root branching number, and soil physicochemical properties significantly affected plant biomass, root diameter and root branching number, and the interaction of the three factors affected biomass and root diameter (**Table 4**). In terms of plant biomass, at the Shaanxi site it was clearly lower than at other sites. Plant biomass of DS993 was clearly higher than that of other genotypes, while plant biomass of DS996 was the lowest (**Figure 1A**). Plant height of *S. miltiorrhiza* in Shaanxi and Sichuan was relatively low, and plant height of DS993 was higher than that of other genotypes except Shandong, while plant height of DS996 was the lowest in all samples except Anhui (**Figure 1B**). Root diameter of *S. miltiorrhiza* in Anhui was higher than in Shandong, Shaanxi and Sichuan, but had no significant difference with that in Beijing. Root diameter of DS993 in Beijing, Shaanxi and Sichuan was higher than that of other genotypes, whereas the root diameter of DS996 was the smallest among all the samples (**Figure 1C**). Moreover, root branching number in Shandong was lower than that in other sample sites. Root branching number of DS993 was higher than other genotypes in Beijing, Shaanxi and Sichuan, and DS2000 in Shandong was the greatest (**Figure 1D**). In conclusion, the biomass and morphological parameters of *S. miltiorrhiza* growing in Shaanxi was significantly lower than in other producing areas. DS993 has the best growth in all production areas, followed by DS2000, and DS996 has the worst growth.

**Table 4:** Three-way ANOVA of the effect of genotype, climatic factors and soil physicochemical properties on the performance and active ingredient contents of *Salvia miltiorrhiza*.

Index	Genotype		Climate		Soil		Genotype*Climate		Genotype*Soil		Climate*Soil		Genotype*Climate*Soil	
	*F*	*P*	*F*	*P*	*F*	*P*	*F*	*P*	*F*	*P*	*F*	*P*	*F*	*P*
Plant biomass (g)	7.24	**	5.05	*	9.26	**	3.29	NS	0.89	NS	4.82	NS	5.98	*
Plant height (cm)	6.97	*	4.10	NS	2.74	NS	2.28	NS	3.16	NS	2.06	NS	3.22	NS
Root diameter (mm)	8.22	**	4.86	NS	7.10	**	5.15	*	1.05	NS	1.33	NS	6.24	*
Root branching number (No.)	7.56	**	5.81	*	5.88	*	5.03	*	2.20	NS	3.47	NS	4.51	NS
Tanshinone ι content (%)	5.79	*	5.62	*	1.03	NS	2.19	NS	0.28	NS	6.66	*	7.25	**
Tanshinone IIA content (%)	6.12	*	5.29	*	5.06	*	2.41	NS	1.55	NS	5.23	*	6.02	*
Cryptotanshinone content (%)	4.58	NS	6.53	*	6.12	*	3.28	NS	2.29	NS	4.18	NS	2.13	NS
Rosmarinic acid content (%)	4.63	NS	5.78	*	7.69	**	2.21	NS	1.68	NS	3.06	NS	1.06	NS
Salvianolic acid B content (%)	5.27	*	6.80	*	7.53	**	5.05	*	0.83	NS	2.22	NS	4.81	NS

*indicates the significant difference at P < 0.05. **indicates the significance difference at P < 0.01. NS, means no significant difference.

### Active ingredient contents

**Figure 2:**
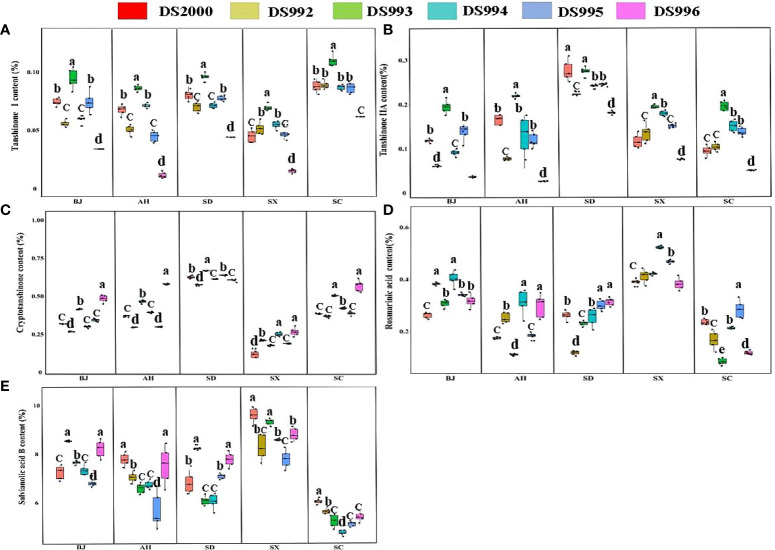
Active component contents of *Salvia miltiorrhiza* genotype in different cultivation locations. Tanshinone I content **(A)**; TanshinoneIIA content **(B)**; Cryptotanshinone content **(C)**; Rosmarinic acid content **(D)**; Salvianolic acid B content **(E)**. BJ, Beijing; AH, Anhui; SD, Shandong; SX, Shaanxi; SC, Sichuan. Different letters above the error bars indicate significant difference at *P* < 0.05.

Three-way ANOVA results showed that genotypes had significant effects on the contents of tanshinone I, tanshinone IIA and salvianolic acid B; climatic factors had significant effects on the contents of tanshinone, cryptotanshinone, rosmarinic acid and salvianolic acid B; soil physicochemical properties had significant effects on the contents of tanshinone IIA, cryptotanshinone, rosmarinic acid and salvianolic acid B. However, the interaction of the three factors only affected the contents of tanshinone I and Tanshinone IIA (**Table 4**). The content of tanshinone I in Sichuan was higher than in other sample sites, and lower in Shaanxi (**Figure 2A**). The tanshinone IIA content in Shandong was higher than in other sample sites (**Figure 2B**). The content of cryptotanshinone in Shandong was higher than in other sample sites, but it was the opposite in Shaanxi (**Figure 2C**). The rosmarinic acid content in Shaanxi was higher than in other sample sites (**Figure 2D**). The content of salvianolic acid B in Shaanxi was higher than in other sample sites, but was lowest in Sichuan (**Figure 2E**).

In terms of the genotype, the content of tanshinone ι in DS993 was higher than that of other genotypes at all sample sites, and lowest in DS996 (**Figure 2A**). The tanshinone IIA content in DS993 was higher than in other genotypes except in Shandong, and the content of tanshinone IIA in DS996 was lowest at all sample sites (**Figure 2B**). The content of cryptotanshinone in DS996 was higher than that of other genotypes except in Shandong, Shaanxi and Sichuan (**Figure 2C**). For rosmarinic acid, DS994 had the highest content in Shaanxi, DS995 and DS996 had the highest content in Shandong, while DS995 had the highest content in Sichuan (**Figure 2D**). For salvianolic acid B, the contents of DS992 and DS996 were higher than that of other genotypes in Beijing, DS2000 and DS996 were the highest in Anhui, DS992 and DS996 was the highest in Shandong, and DS2000 was higher than that in other genotypes in Sichuan (**Figure 2E**).

In conclusion, the lipid-soluble composition (tanshinone I, tanshinone IIA, and cryptotanshinone) of Shandong Province is significantly higher than that of other provinces. The water-soluble components (rosmarinic acid, and salvianolic acid B) of Shaanxi Salvianolic acid were significantly higher than those of other areas. DS993 has the highest content of tanshinone I and tanshinone IIA, and DS996 has the highest content of cryptotanshinone, while the water-soluble component has no genotype preference, which is more influenced by origin factors.

### Soil physicochemical properties

**Figure 3:**
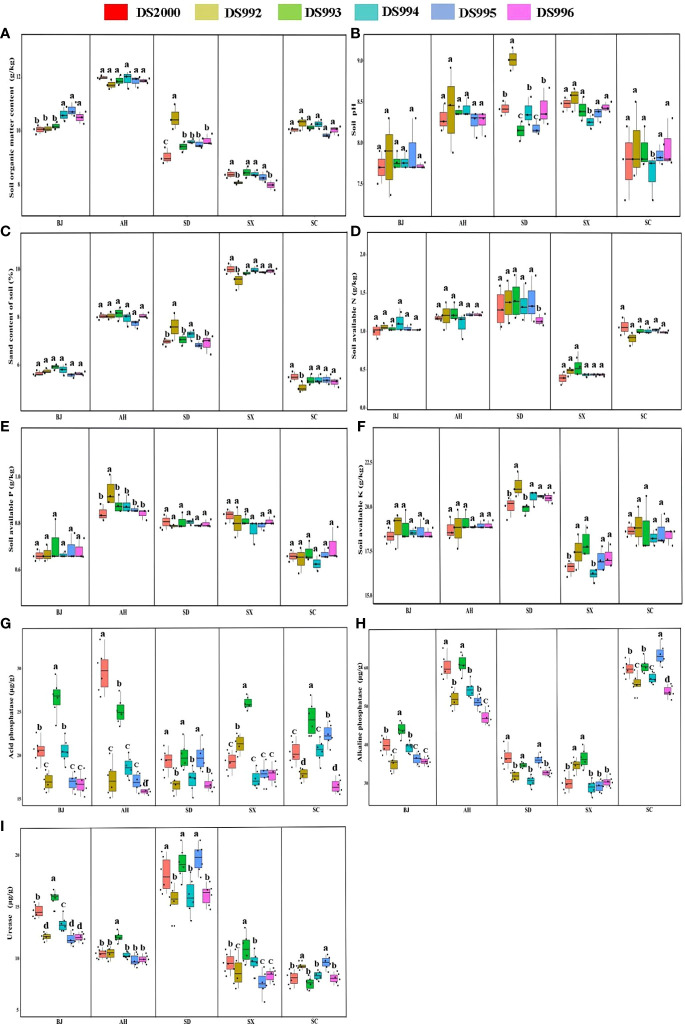
Rhizosphere soil physicochemical properties of *Salvia miltiorrhiza* genotype in different cultivation locations. Soil organic matter content **(A)**; Soil pH **(B)**; Soil sand content **(C)**; Soil available N **(D)**; Soil available P **(E)**; Soil available K **(F)**; Soil acid phosphatase **(G)**; Soil alkaline phosphatase **(H)**; Soil urease **(I)**. BJ: Beijing; AH: Anhui; SD, Shandong; SX, Shaanxi; SC, Sichuan. Different letters above the error bars indicate significant difference at *P* < 0.05.

Plant growth and development are closely related to soil physicochemical properties. The results showed that soil organic matter content was highest in Anhui and lowest in Shaanxi, but there was no significant difference between Beijing, Shandong and Sichuan. The content of soil organic matter of the DS992 rhizosphere was higher than that of other genotypes in Shandong (**Figure 3A**). Soil pH in Anhui, Shandong and Shaanxi were higher than the other sample sites, and soil pH in the DS992 rhizosphere was higher than that of other genotypes in Shandong (**Figure 3B**). In addition, the highest sand content was found in the Shaanxi soil, and it was lower in Beijing and Sichuan than in the other sample sites (**Figure 3C**). The content of soil available N in Shaanxi was lower than in other sample sites, and there was no significant difference between the other sites (**Figure 3D**). The content of soil available P in Anhui, Shandong and Shaanxi was higher than in Beijing and Sichuan (**Figure 3E**). In terms of soil available K, Shandong had the highest and Shaanxi had the lowest. The content of soil available K under DS992 was higher than under other genotypes in Shandong, while the content of soil available K from DS994 was lower than under other genotypes in Shaanxi (**Figure 3F**). For soil phosphatase activity, there was no significant difference among different sample sites except for some individual sites. The higher activity of soil acid phosphatase from DS993 occurred in Beijing, Shaanxi and Sichuan, while the higher activity of soil acid phosphatase from DS2000 occurred in Anhui (**Figure 3G**). Soil alkaline phosphatase activity in Anhui and Sichuan was higher than in other sample sites (**Figure 3H**). For soil urease activity, it was clearly higher in Shandong than other sample sites, and was higher from DS993 than other genotypes in Beijing,Anhui and Shaanxi (**Figure 3I**).

In conclusion, rhizosphere soil organic matter, pH and nutrient indexes of *S.miltiorrhiza* were mainly regulated by environmental factors in different producing areas, while soil enzyme activities were synergically affected by genotype and environmental factors.

### Soil microbial composition

**Figure 4:**
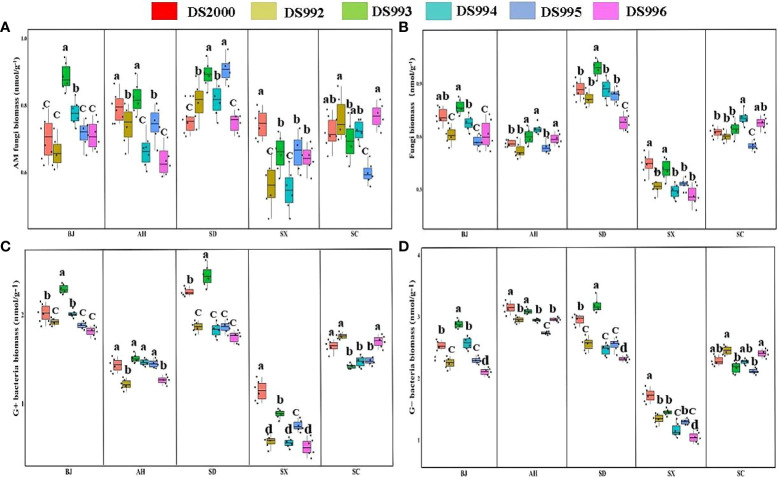
Rhizosphere soil microbial composition of *Salvia miltiorrhiza* genotype in different cultivation locations.Arbuscular mycorrhizal (AM) fungi biomass **(A)**; Fungi biomass **(B)**; G+ bacteria biomass **(C)**; G- bacteria biomass **(D)**. BJ, Beijing; AH, Anhui; SD, Shandong; SX, Shaanxi; SC, Sichuan. Different letters above the error bars indicate significant difference at *P* < 0.05.

Three-way ANOVA showed that genotypes significantly affected G+ and G- bacteria contents, climatic factors and soil physicochemical properties significantly affected arbuscular mycorrhizal (AM) fungi, fungi, G+ and G- bacteria contents, and the interaction of the three factors significantly affected soil fungi, G+ and G- bacteria contents (**Table 5**). In terms of producing areas, the AM fungal biomass in Shaanxi was lower than that in other plots. In terms of genotypes, Beijing DS993 had the highest AM fungal biomass, and Shaanxi DS2000 had the highest AM fungal biomass (**Figure 4A**). The biomass of soil fungi was highest in Shandong, and lowest in Shaanxi. It was higher under DS993 than other genotypes in Shandong, and was lowest from DS996 in Shandong (**Figure 4B**). The higher biomass of G+ bacteria was in Beijing and Shandong, and the lowest was in Shaanxi. The biomass of G+ bacteria from DS993 was the higher in Beijing and Shandong, while this value from DS2000 was highest at the Shaanxi site (**Figure 4C**). In terms of G- bacteria, the biomass was lower in Shaanxi than in other sample sites. G- bacteria from DS993 was higher in Beijing and Shandong, and the highest biomass of G- bacteria from DS2000 was in the Shaanxi site (**Figure 4D**).

**Table 5:** Three-way ANOVA of the effect of genotype, climatic factors and soil physicochemical properties on microbial composition of *Salvia miltiorrhiza*.

Index	Genotype		Climate		Soil		Genotype*Climate		Genotype*Soil		Climate*Soil		Genotype*Climate*Soil	
	*F*	*P*	*F*	*P*	*F*	*P*	*F*	*P*	*F*	*P*	*F*	*P*	*F*	*P*
AM fungi (nmol/g^-1^)	4.77	NS	5.39	*	6.28	*	4.26	NS	1.29	NS	6.03	*	4.55	NS
Fungi (nmol/g^-1^)	4.32	NS	7.02	**	5.19	*	5.14	*	2.36	NS	3.52	NS	6.07	*
G+ bacteria (nmol/g^-1^)	5.89	*	6.14	*	8.09	**	5.22	*	6.10	*	5.18	*	8.24	**
G- bacteria (nmol/g^-1^)	6.08	*	6.38	*	8.43	**	5.60	*	4.03	NS	2.22	NS	5.86	*

*indicates the significant difference at P < 0.05. **indicates the significance difference at P < 0.01. NS, means no significant difference.

In conclusion, the rhizosphere soil microbial content of *S. miltiorrhiza* in Shaanxi was significantly lower than that of other producing areas, while that of Shandong soil was generally higher. In additions, the rhizosphere soil microbial content of DS993 was the highest in most production areas, while DS996 was the opposite.

### Variation partitioning of growth parameters

**Figure 5:**
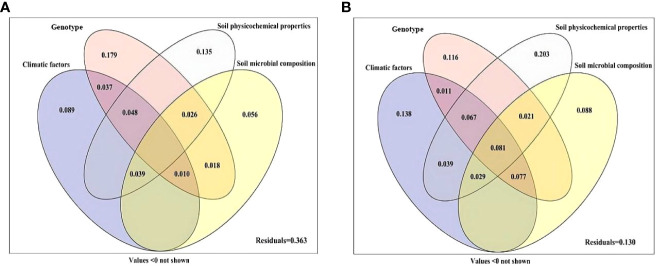
Variation partitioning of climatic factors, genotype, soil physicochemical properties and soil microbial composition on the performance **(A)** and active ingredient **(B)** of *S. miltiorrhiza*. Climatic factors (including average temperature, mean temperature difference, soil mean temperature and mean rainfall); Genotype (including DS2000, DS992, DS993, DS994, DS995 and DS996); Soil physicochemical properties (including soil pH, organic matter, sand content, available N, available P, available K, acid phosphatase, alkaline phosphatase and urease); Soil microbial composition (AM fungi, fungi, G+ bacteria and G- bacteria).

Variance partitioning analysis was performed to quantify the contribution of genotype, climatic factors, soil physicochemical properties and soil microbial composition to plant growth and active ingredient content (**Figure 5**). The combination of genotype, climatic factors, soil physicochemical properties and soil microbial composition explained 63.7% of the variation in the growth parameters (**Figure 5A**), and 87.0% of the variation in active ingredient content (**Figure 5B**). The genotype was the most important factor affecting the growth parameters of *S. miltiorrhiza*, with a single explanation of 17.9%, and the individual explanations of climatic factors, soil physicochemical properties and soil microbial composition were 8.9%, 13.5% and 5.6%, respectively (**Figure 5A**). For active ingredient content, soil physicochemical properties were the most important factors affecting the active components of *S. miltiorrhiza*, and the single explanation reached 20.3%. The individual explanations of climatic factors, genotype and soil microbial composition were 13.8%, 11.6% and 8.8%, respectively. The interaction effect of the above four main factors was 8.1% (**Figure 5B**).

### Correlation analyses

**Figure 6:**
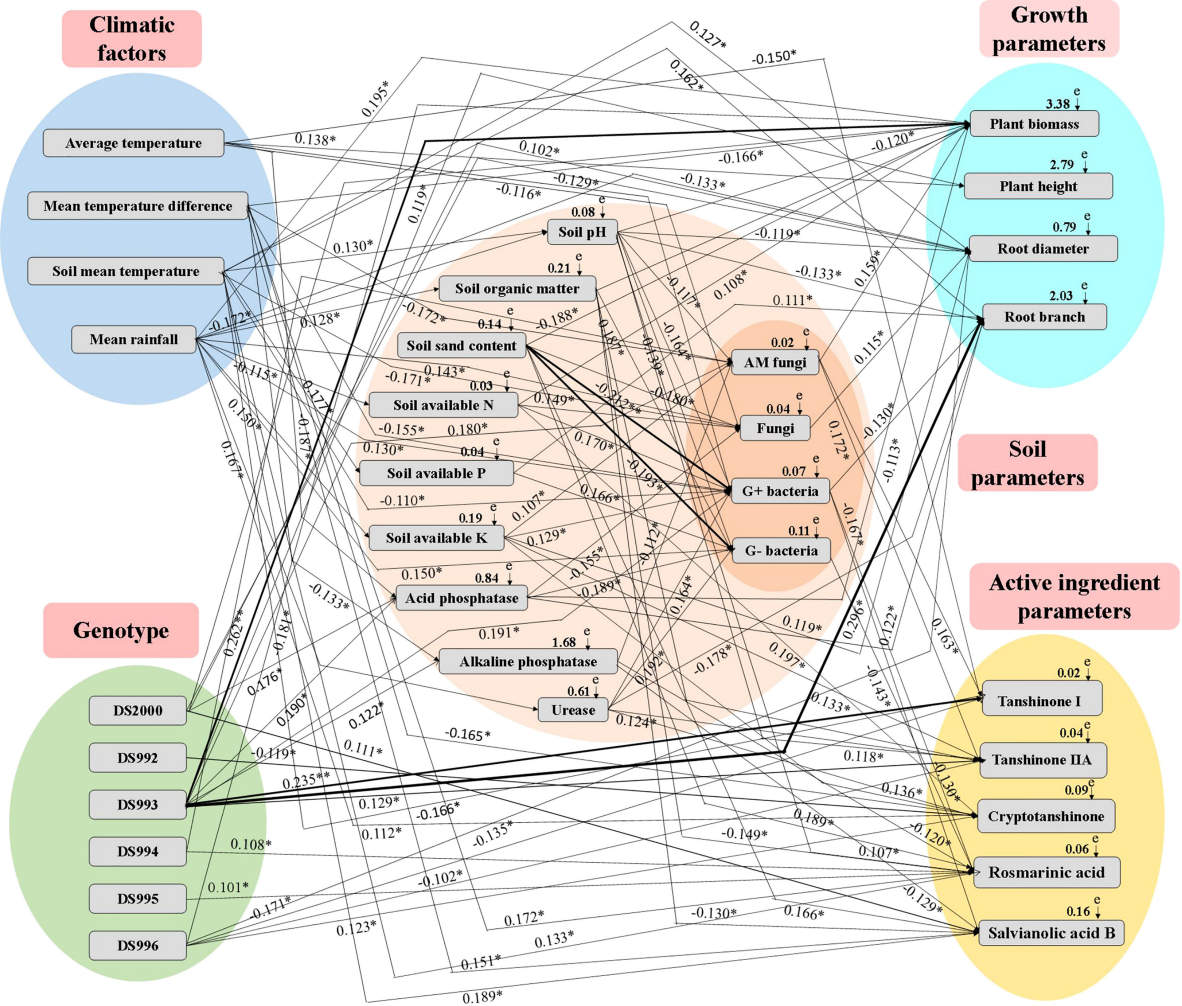
Structural equation model (SEM) showing the causal relationships among climatic factors, genotype, soil physicochemical properties, microbial composition, growth parameters and active ingredients. The final model fitted the data well: maximum likelihood, *X^2 =^* 105.89, *df*=18, *P*=0.001, GFI=0.925, AIC=167.229, and RMSEA=0.371. Solid lines and dashed lines indicate significant and non-significant pathways, respectively. The width of the solid lines indicates the strength of the causal effect, and the numbers near the arrows indicate the standardized path coefficients (*P < 0.05, **P < 0.01).

The Mantel test and SEM revealed significant relationships between climatic factors, genotype, soil properties, growth parameters and active components (**Supplementary Table S1** and **Figure 6**). The relative effects of climatic factors, genotype and soil properties on growth parameters and active components were quantitatively determined using the correlation coefficient (*R*-value) and SEM (*X* ^2 =^ 105.89, *df*=18, *P*=0.001, GFI=0.925, AIC=167.229, and RMSEA=0.371). Specifically, average temperature positively affected plant height, but negatively affected soil acid phosphatase, fungi, G+ bacteria and tanshinone I. Mean temperature difference positively affected acid phosphatase, rosmarinic acid and salvianolic acid B, and negatively affected soil fungi, G+ bacteria, total biomass and cryptotanshinone. Soil mean temperature positively affected soil pH, available P, and root diameter, root branching and rosmarinic acid, and negatively affected soil fungi. Mean rainfall positively affected soil organic matter, available K, urease, fungi, G+ bacteria and G- bacteria, total biomass and cryptotanshinone; and negatively affected soil pH, available N, plant height and root diameter. DS2000 positively affected soil acid phosphatase and root diameter. DS992 negatively affected cryptotanshinone. DS993 positively affected soil acid phosphatase, alkaline phosphatase, AM fungi, G+ bacteria, plant biomass, plant height, root diameter, root branching number, tanshinone I and tanshinone IIA. DS994 and DS995 both positively affected rosmarinic acid, while DS996 positively affected cryptotanshinone, and negatively affected plant biomass, tanshinone ι and tanshinone IIA. Soil pH positively affected soil sand content and tanshinone IIA, but negatively affected soil available P, acid phosphatase, AM fungi, G+ bacteria, G- bacteria, plant biomass, root diameter and root branching number. Soil organic matter positively affected G- bacteria, plant biomass and tanshinone IIA; it negatively affected rosmarinic acid and salvianolic acid B. Soil sand content positively affected rosmarinic acid, and negatively affected fungi, G+ bacteria and G- bacteria, as well as plant biomass and tanshinone IIA. Soil available N positively affected fungi, G+ bacteria, plant biomass and cryptotanshinone. Soil available P positively affected G- bacteria, and root branching number. Soil available K positively affected AM fungi, G+ bacteria, root diameter and tanshinone IIA, but negatively affected rosmarinic acid. Soil acid phosphatase positively affected plant biomass and root diameter, and negatively affected soil fungi and G- bacteria. Soil alkaline phosphatase negatively affected AM fungi, rosmarinic acid and salvianolic acid B. Soil urease positively affected G+ bacteria, G- bacteria, and tanshinone IIA and cryptotanshinone, but negatively affected root branching number. AM fungi positively affected plant biomass, tanshinone I and tanshinone IIA. Fungi positively affected root diameter. G+ bacteria negatively affected root branching number, rosmarinic acid and salvianolic acid B. G- bacteria negatively affected rosmarinic acid.

In conclusion, the most active climate factor is average rainfall, which has the greatest influence on plant growth, effective components and soil parameters in the system of multi-factor synergistic regulation of growth and accumulation of active components of *S. miltiorrhiza*. DS993 was the most active genotype factor, and its regulation of plant growth, active components, soil enzyme activities and microbial content had the most positive effects, which was consistent with the phenomenon that this genotype grew well in most producing areas. Among the soil factors, soil organic matter, available nitrogen and AM fungi factors had the greatest effect on the growth and development of *S. miltiorrhiza*.

## Discussion

### Effect of genotype and climatic factors on growth and active ingredient of *S miltiorrhiza*

The current study has confirmed that there were large variations in the plant biomass, morphological parameters and active component content of six genotypes of *S. miltiorrhiza* in five cultivation locations. The SEM analysis and variance partitioning showed that genotype was the primary factor affecting the growth parameters, and also contributed significantly to the content of active components, accounting for 11.6%. Among the six genotypes, the growth traits and contents of liposoluble components such as tanshinone ι and tanshinone ιIA in DS993 were higher, whereas those of DS996 were lower. Moreover, higher contents of the water-soluble compounds were found in DS2000 (salvianolic acid B), DS994 and DS995 (rosmarinic acid). Previous studies have also indicated that different genotypes showed clear differences in plant height, number of flowers and biomass of *Anthemis* species (Alizadeh and Jafari, 2016), as well as growth and flavonoid accumulation in *Cyclocaryapaliurus* (Liu et al., 2016). Variations in morphological features and apo-carotenoid content among corms of different genotypes has also been reported by other authors (Baghalian et al., 2010; Ghanbari et al., 2019). Climate change is, without doubt, a direct and important factor affecting the growth, secondary metabolites and distribution of medicinal plants. For instance, in our study, average temperature positively affected plant height, and mean temperature difference negatively affected plant biomass. Soil mean temperature positively affected root diameter and root branching number, whereas mean rainfall positively affected plant biomass and negatively affected root diameter. Climate change can directly or indirectly affect the growth, development, physiological metabolism, accumulation of active components, yield and quality of medicinal plants (Thakur and Kumar, 2021; Patni et al., 2022). In our study, the pure variability of active ingredients accounted for by climatic factors was 13.8%. In particular, temperature difference, soil temperature and rainfall had significant effects on the cryptotanshinone, rosmarinic acid and salvianolic acid B content. Response of growth and active ingredients to genotypes and climatic factors are very important to help determine the best cultivation areas for medicinal plants in the future (Zhang et al., 2019; Karalija et al., 2022).

### Effect of genotype and climatic factors on rhizosphere soil physicochemical properties and microbial composition under *S. miltiorrhiza*

The results showed that the rhizosphere soil physicochemical properties and microbial composition under *S. miltiorrhiza* were more affected by climatic factors than by the genotype grown. For example, climatic factors significantly affect all soil physicochemical properties except soil sand content and acid phosphatase. Climate is a factor that has a profound impact on plant growth and soil properties, especially temperature and precipitation, which affect soil formation and evolution, nutrient cycling and soil bioactivity (Heydari et al., 2019). We also found that some soil properties were influenced by climatic conditions. For example, air temperature significantly affected acid phosphatase activity; soil temperature positively affected soil pH and available P content; and rainfall positively affected soil organic matter, available K content and urease activity, while soil pH and available N were negatively affected. Currently, although studies have shown that climatic condition such as temperature and precipitation have direct effects on soil microbes (Donhauser and Frey, 2018; Ren et al., 2018), the indirect effects of climatic condition on soil microbes still need to be explored. Our results provide evidence that climate factors directly or indirectly affect the soil microbial composition and biomass. In general, air temperature directly affected soil fungi and G+ bacterial biomasses, and indirectly affected the biomass of these microbial components by affecting acid phosphatase activity. Soil temperature directly affected soil fungal and G+ bacterial biomasses, and indirectly affected soil G+ bacterial biomass by affecting soil pH. Rainfall directly affected soil fungi, G+ and G- bacterial biomasses, and indirectly affected the biomass of these microbial components by affecting soil pH, organic matter, available N, available K and urease. Zhou et al. (2018) and Ren et al. (2018) found that rainfall variation can affect soil microbial biomass both directly (by, for example, changing soil moisture) and indirectly (by, for example, modification of plant primary productivity). Freedman et al. (2021) reported that rainfall and temperature directly affect microbial classification and diversity; and climate variables also indirectly affect soil microorganisms through plant growth. In addition, G+ bacteria and G- bacteria contain features of oligotrophic (nutrient-poor) and cotrophic (nutrient-rich) microorganisms, respectively, and increasing temperatures enhanced fast-growing (cotrophic) bacteria that prefer high nutrient availability (Männisto et al., 2016; Naylor and Coleman-Derr, 2017). Numerous results have shown that global warming has a direct effect on soil microbial composition and activity, and also alters soil microbial community structure indirectly by affecting plant photosynthesis, respiration and root exudation (Wu and Yu, 2019).

Similarly, the influence of genotypes on the physicochemical properties and microbial communities of rhizosphere soil of *S. miltiorrhiza* should not be ignored. We found that different genotypes had significant effects on soil acid phosphatase and alkaline phosphatase activities as well as G+ and G- bacterial biomass.Interestingly, soil pH, organic matter and available K under DS992 were higher than those from the other genotypes in the Shandong site, while the activity of acid phosphatase, alkaline phosphatase, and urease, along with the biomass of AM fungi and G+ bacteria under DS993 at the Beijing site were higher than those of other genotypes. This may be related to the root distribution and exudates from genotypes with different genetic backgrounds, occurring in a given location associated with regulating a particular soil nutrient status. Plant root distribution can influence soil physicochemical properties such as substrates and pH along soil profiles (Pietri and Brookes, 2008). Plant root systems are the main medium for exudates into rhizosphere soil, and root exudates can change the physicochemical properties and microbial composition of the rhizosphere soil to a plant’s benefit (Bakker et al., 2018; Shi et al., 2021). Zhang et al. (2022) reported that ginseng rhizosphere bacterial community structure is affected by genotype, and there is a correlation between ginseng genotypes and the bacterial population structure. Understanding soil biota, abiotic soil environment and genotypic effects can, therefore, help to elucidate the ecological adaptability of different genotypes (De Long et al., 2019).

### Effect of rhizosphere soil physicochemical properties and microbial composition on growth and active ingredients in *S miltiorrhiza*

Plants living in soil inevitably interact directly and indirectly with soil biotic and abiotic components. The SEM analysis and variance partitioning showed that soil physicochemical properties directly influenced plant growth and are the primary factor affecting the active ingredients content of *S. miltiorrhiza*. They also contributed significantly to the growth parameters, accounting for 13.5%. However, individual soil factor effect obviously differed. For example, soil pH positively affected tanshinone IIA, and negatively affected total biomass, root diameter and root branching number. Soil organic matter increased plant biomass and tanshinone IIA content, but reduced rosmarinic acid and salvianolic acid B contents. Soil acid phosphatase increased root branching number and root diameter, but reduced plant biomass. Zhalnina et al. (2014) considered that soil pH changed the effectiveness of plant nutrition by controlling the chemical form of soil compounds, thereby indirectly regulating the soil microbial community and plant growth. It has been shown that soil enzymes can affect plant growth by changing the soil chemical state (Adetunji et al., 2017). Mi et al. (2020) found that the total sugar, reducing sugar, betaine, flavones and amino acids were clearly different in *Lycium barbarum* from different regions, but that mean air temperature, annual precipitation and soil factors affecting different chemical compounds varied. Mykhailenko et al. (2020) found a moderate positive correlation between the amount of p-cumaric acid and soil N, but also a moderate negative correlation between the amount of hyperoside and soil P in the rhizome of *Iris* spp. de Albergaria et al. (2021) reported that soil fertility affected the total phenol content of *Cenostigma microphyllum* negatively, but had no significant impact on total tannin content. In addition, soil texture, nutrients, temperature and other characteristics have been shown to significantly affect the growth and distribution of plant roots (Buras et al., 2020). These results indicate that the performance and quality of cultivated medicinal plants are strongly affected by growth conditions such as temperature, light and soil nutrient status.

At present, only a few studies have tested the indirect effects of soil factors on plant performance in different cultivation areas.Therefore, we studied the changes of soil microbial content, hoping to more completely show the cooperative regulation of different plant genotypes and environmental factors on soil microbial flora, so as to achieve the purpose of affecting plant growth and accumulation of active components. Rhizospheres hold huge, diverse soil microbial communities, and these microorganisms may be beneficial, neutral or harmful to the growth and development of plants. In particular, beneficial microorganisms (AM fungi, plant growth promoting rhizobacteria, actinomycetes, etc.) can effectively promote plant growth, development and health by improving disease resistance, maintaining more water, or absorbing and using more nutrients (Olanrewaju et al., 2019). In our study, variance partitioning showed that soil microorganisms contributed significantly to the growth parameters and active ingredients of *S. miltiorrhiza*, accounting for 5.6% and 8.8% respectively. On the one hand, AM fungi directly and positively affected plant biomass, tanshinone I and tanshinone IIA contents. Fungi directly and positively affected root diameter. Studies have confirmed that the mycelial network of AM fungi expand the space of mineral and water absorption in root system, which promotes the growth and component accumulation of medicinal plants (Sun et al., 2021). G+ bacteria directly and negatively affected root branching number, rosmarinic acid and salvianolic acid B contents, but G- bacteria only directly and negatively affected rosmarinic acid content. Plant growth-promoting rhizosphere bacteria play a beneficial role in plant growth by affecting hormone regulation, nutrient uptake and utilization, as well as nitrogen fixation (Vafadar et al., 2014). Chen et al. (2021) found that Actinobacteria and Chloroflexi in a *Sophora flavescens* rhizosphere were associated with the total accumulation of matrine and oxymatrine, and that the Actinobacteria phylum, in particular, had a direct effect their accumulation. On the other hand, soil physicochemical properties indirectly affected the growth and active ingredients by modifying soil microorganisms. For instance, in this study, soil pH negatively combine with soil available N, available K and urease positively indirectly inhibited root branch and water-soluble component (rosmarinic acid and salvianolic acid B) content by modifying soil G+ bacteria; soil organic matter, available P and urease positively combine with soil pH, and acid phosphatase negatively indirectly inhibited rosmarinic acid content by modifying soil G- bacteria. Santoyo et al. (2017) reported that soil pH, temperature, soil type, geographical factors and climatic conditions may all play a key role in modulating soil microbial diversity, as well as having an indirect impact on plant health and development. Haas et al. (2018) suggested that soil microbial composition depends on soil nutrient status as well as the quantity and quality of root exudates, thus affecting plant nutrition and physiological metabolism (Xie et al., 2019). Liu et al. (2022) found that soil chemical properties influenced the microbial composition of rhizosphere soil, and thus contribute to the growth and development of *Bupleurum chinense*. These findings may provide new perspectives for promoting the development of medicinal plant cultivation by improving soil microbial composition (Yuan et al., 2022).

## Conclusion

In this study, we confirmed that the production performance, active component content, rhizosphere soil physicochemical properties and microbial composition were sensitive to different genotypes of *S. miltiorrhiza* and climatic factors. Genotype and climatic factors had both direct and indirect effects (changing soil physicochemical properties, microbial composition and biomass). We found that plant genotype and soil physicochemical properties were the primary factors affecting the performance, whereas plant genotype, climatic factors and soil physicochemical properties mainly contribute to the active components content of *S. miltiorrhiza*. In addition, the gram-positive and gram-negative bacterial biomasses were clearly affected by the plant genotype, and all the tested microbial group biomasses were affected by climatic factors. Thus, we suggest that modifying soil microbial composition could be valuable to optimize the cultivation of *S. miltiorrhiza*. The data obtained suggest a complex relationship between climate, genotype, soil properties, growth and active ingredients content, but highlight that selecting a suitable genotype and cultivation area is crucial for maximizing yield and quality of *S. miltiorrhiza.* In summary, DS993 was the most suitable genotype for the five production areas, both in terms of growth traits and medicinal components. In terms of production areas, DS993 and DS2000 were suitable for planting in Shandong Province. DS996 is not suitable for all the above production areas.

## Data availability statement

The original contributions presented in the study are included in the article/**Supplementary Material**. Further inquiries can be directed to the corresponding authors.

## Author contributions

CH, DL and XL conceived and designed the experiments and wrote the paper. CH,TH and CL performed the experiments. CH, TH, and PS analyzed the data. All authors contributed to the article and approved the submitted version.
