# Supplementary material for: Deciphering the effects of genotype and climatic factors on the performance, active ingredients and rhizosphere soil properties of Salvia miltiorrhiza
Source: Front Plant Sci. 2023 Apr 20;14:1110860. doi: 10.3389/fpls.2023.1110860 (PMC10157250; doi:10.3389/fpls.2023.1110860)
Supplement: Supplementary file 1 [file Table_1.docx]

Table S1 Mantel tests showing correlationships (R values) between climate, genotype, soil physicochemical [properties](https://fanyi.so.com/#properties), microbial composition, plant growth parameters and active components.

Note:The*symbol indicates the significant difference, P<0.05. The** symbol indicates the significance level intervenes between P<0.01. At,Average temperature;Mtd,Mean temperature difference;Smt,Soil mean temperature;Mr,Mean rainfall;Sph,Soil pH;Som,Soil organic matter;Sac,Soil sand content;San,Soil available N;Sap,Soil available P;Sak,Soil available K;Acp,Acid phosphatase;Alp,Alkaline phosphatase;Ur,Urease;Am,AM fungi;G+,G+ bacteria;G-,G- bacteria;Pb,Plant biomass;Ph,Plant height;Rd,Root diameter;Rb,Root branch;Tsh I,Tanshinone I ;TshⅡA,TanshinoneⅡA;Cpt,Cryptotanshinone;Ra,Rosmarinic acid;Sab,Salvianolic acid B.

,
